# Remodeling of Oncogene-Related Molecular Networks Following Bariatric Surgery: a Multi-Omics Study

**DOI:** 10.1007/s11695-026-08959-w

**Published:** 2026-10-05

**Authors:** Mohammed Salhi, Sarah Al-Shahabi, Summaiya Khatoon, Aaiza Asif, Marwa Naeem, Muhammad Enamul Hoque Chowdhury, Shona Pedersen, Fakhar Shahid, Muhammad Jawad Khan, Zumin Shi, Aisha Naeem

**Affiliations:** 1 Qatar University, Doha, Qatar; 2 COMSATS University Islamabad, Islamabad, Pakistan; 3 Hamad Medical Cooperation, Doha, Qatar; 4 Doha, Qatar

**Keywords:** Metabolic bariatric surgery, Obesity, Cancer risk, Transcriptomics, Proteomics, Metabolomics

## Abstract

Obesity is a well-established risk factor for multiple cancers, and Metabolic Bariatric Surgery (MBS) has been associated with reduced cancer incidence and mortality. However, the molecular mechanisms underlying this risk reduction remain incompletely understood. Many recent transcriptomic, proteomic, and metabolomic studies have enabled system-wide molecular profiling following MBS. We performed a systematic analysis of publicly available MBS-related omics datasets, including transcriptomic (*n* = 22), proteomic (*n* = 6), and metabolomic studies (*n* = 5), with pre- and post-operative comparisons. Comparative molecular analyses across all omics datasets were performed using Ingenuity Pathway Analysis (IPA). Sustained suppression of key oncogenic pathways, including Molecular Mechanisms of Cancer, Role of Tissue Factor in Cancer, and metastasis-associated signaling, was observed in tissue transcriptomic datasets (adipose, skeletal muscle, and gastrointestinal tract). In contrast, transcriptomic datasets predominantly showed immune remodeling, characterized by activation of innate immune pathways. Key molecular markers, including *CCL2*, *EGFL6*, and S100A8, were identified as consistently regulated across multiple datasets. Proteomic analyses revealed early postoperative suppression of inflammatory and acute-phase proteins, progressive downregulation of complement and coagulation cascades, and sustained upregulation of endocrine regulators such as sex hormone-binding globulin and extracellular matrix–stabilizing factors. Metabolomic profiling demonstrated significant alterations in amino acid, bile acid, and lipid metabolism following MBS. These molecular changes collectively reflect a shift toward a less inflammatory, hormonally balanced, and metabolically favorable systemic environment. These findings highlight the impact of MBS in inhibiting cancer-promoting biological processes and identify candidate molecular biomarkers for future translational and oncologic research.

**Supplementary Information:**

The online version contains supplementary material available at https://doi.org/10.1007/s11695-026-08959-w.

## Introduction

 Obesity is known to be associated with serious comorbidities such as cardiovascular diseases and type 2 diabetes mellitus (T2DM) [1]. However, beyond its well-documented cardiometabolic and other health consequences [1], obesity is now recognized as a major independent risk factor for cancer development, progression, and mortality [2–8]. For example, Chlebowski et al. [6] and Guh et al. [7] reported increased cancer risk associated with obesity, including higher breast cancer risk and mortality [6] and increased incidence of most cancer types [7].

Several mechanisms have been proposed to explain the link between obesity and cancer. One probable mechanism involves excess estrogen production by hypertrophied adipose tissue, which directly contributes to an increased risk of hormone-sensitive cancers, including endometrial cancer [9]. Obesity-induced insulin resistance leads to systemic hyperinsulinemia and elevated levels of insulin-like growth factor-1 [10]. These growth factors are potent activators of pro-survival and proliferative signaling pathways and are associated with increased risks of colorectal, thyroid, breast, prostate, ovarian, and endometrial cancers [10, 11].

Metabolic Bariatric Surgery (MBS) represents the most effective and durable intervention for inducing and sustaining significant weight loss in patients with severe obesity [12]. Notably, a recent meta-analysis has demonstrated an association between MBS and reduced incidence and mortality in several obesity-related cancers [13]. However, despite strong epidemiological evidence, the mechanisms linking surgical weight loss to reduced cancer risk remain insufficiently characterized. Therefore, understanding the underlying molecular mechanisms, networks, and associated biological pathways that contribute to the reduction in cancer risk following this intervention is critical.

Beyond its well-established clinical benefits, multiple high-throughput transcriptomic, proteomic, and metabolomic studies indicate that MBS induces broad and sustained molecular remodeling across multiple tissues [14–16]. These omics studies are particularly relevant given the well-established links between obesity, chronic inflammation, and increased cancer risk [17, 18]. Molecular changes induced by the weight loss following MBS may therefore represent key biological mechanisms underlying the reduced cancer incidence and improved survival observed in post-surgical patients.

Despite growing interest, molecular evidence following MBS remains fragmented by small sample sizes, heterogeneous tissues and follow-up durations, and platform-specific analytical biases. As a result, it remains challenging to distinguish robust biological signals from study-specific effects or to derive a unified molecular framework explaining the systemic impact of MBS on pathways related to metabolism, inflammation, and cancer biology. This study comprehensively analyzes publicly available multi-omic datasets to identify consistent molecular signatures following MBS and investigate their biological significance, with a focus on cancer-related mechanisms.

## Methods

### Dataset Identification

Publicly available multi-omic datasets examining molecular changes before and after MBS were identified through a structured literature search. Transcriptomic, proteomic, and metabolomic datasets were included if they comprised pre- and post-MBS samples and provided processed or normalized molecular data suitable for downstream analysis.

Studies without pre- and post-MBS comparisons or differential analysis, as well as animal studies and reviews, were excluded. Available demographic and clinical characteristics (age, sex, BMI, surgery type, and sampling time points) were extracted and summarized in Table 1 but not included in the meta-analysis due to heterogeneity and incomplete reporting. Dataset search and selection, MeSH search terms, screening criteria, and the repositories searched are detailed in Supplementary Figs. 1–3.

**Table 1:** Overview of included datasets across tissues for multi-omics analysis following metabolic bariatric surgery (MBS)

Study ID	Year	Age (Years)	BMI (kg/m²)	Tissue/Cell type	Time points (Pre/Post)	*N*	Surgery type	Platform
Transcriptomics								
GSE65540 [39]	2015	37.5 ± 8.3	46.5 ± 5.6	SC AT	Pre; Post (3 m)	44	RYGB; AGB	HTS
GSE29411 [40]	2011	42.7 ± 10	44.5 ± 10.0	AT	Pre; Post (4, 12 m)	15	VGB	HTS
GSE199063 [41]	2022	43 ± 9	43 ± 5	AT	Pre; Post (24, 60 m)	165	RYGB	HTS
GSE66921 [42]	2015	N/A	N/A	SC AT	Pre; Post (24 m)	16	N/A	Microarray
GSE59034 [43]	2014	N/A	> 30	SC WAT	Pre; Post (24 m)	48	RYGB	Microarray
GSE72158 [44]	2015	42.9 ± 10.5	47.1 ± 6.0	SC AT	Pre; Post (12 m)	84	AGB; SG; RYGB	Microarray
GSE53376 [45]	2013	48 ± 10	43.1 ± 4.9	SC AT	Pre; Post (24 m)	32	RYGB	Microarray
GSE137631 [46]	2019	40.0 ± 5.3	48.2 ± 5.0	SM	Pre; Post (9 m)	30	RYGB	HTS
GSE272133 [47]	2024	50.9 ± 4.6	45.9 ± 5.9	SM	Pre; Post (12 m)	52	N/A	HTS
GSE87131 [48]	2016	N/A	N/A	SM	Pre; Post (4 m)	50	RYGB	RT-PCR
GSE5109 [49]	2006	37.0 ± 4.2	60.6 ± 7.7	SM	Pre; Post (12 m)	6	RYGB	Microarray
GSE76762 [50]	2016	N/A	N/A	GM	Pre; Post (3 m)	36	RYGB	Microarray
GSE214758 [51]	2022	N/A	N/A	IE	Pre; Post (6–9 m)	22	RYGB	HTS
GSE113819 [52]	2018	N/A	N/A	Jj	Pre; Post (1 m)	10	RYGB	Microarray
GSE83223 [53]	2016	36.9 ± 10.2	42 ± 5.3	PMN	Pre; Post (6 m)	35	RYGB	Microarray
GSE32575 [54]	2011	39 ± 3	44 ± 1	Monocytes	Pre; Post (3 m)	48	RYGB	Microarray
GSE19790 [55]	2010	50.5 ± 11.9	47.1 ± 11.6	WB	Pre; Post (6–12 m)	22	SG; RYGB	Microarray
GSE271700 [56]	2024	40.2 ± 7	46.5 ± 8.4	PMN	Pre; Post (2, 12 m)	73	SG; RYGB	HTS
GSE66306 [39]	2015	37.5 ± 8.3	46.5 ± 5.6	PMN	Pre; Post (3 m)	38	RYGB; AGB	HTS
GSE126803 [57]	2019	44.7 ± 10.2	46.15 ± 7.32	Breast tissue	Pre; Post (25% weight loss)	60	N/A	HTS
Proteomics								
PXD061410 [58]	2026	≥ 35	≥ 35	SM	Pre; Post (5–6, 12 m)	39	RYGB	LC–MS/MS
PXD057848 [59]	2025	46 ± 10	51 ± 7	Plasma	Pre; Post (9 m)	25	RYGB	LC-ESI-MS/MS
PXD009348 [60]	2018	40–46	≥ 38	Plasma	Pre; Post (1 week, 3 m, 1, 4 years)	47	RYGB	LC–MS/MS
PXD034182 [61]	2025	18–60	35–40	SC AT	Pre; Post (6 m)	61	SG; RYGB	LC–MS/MS
PXD065508 [19]	2025	41–53	≥ 35	GM	Pre; Post (8 m)	12	SG; RYGB	LC–MS/MS
PXD040143 [62]	2025	≥ 18	30–35	Blood/urine	Pre; Post (6 m)	67	Not specified	LC–MS/MS; SWATH-MS
PXD063904 [63]	2026	44.0 ± 12.0	42.9	Urine	Pre; Post (24 m)	16	RYGB; SADI-S; SG	LC–MS/MS
Metabolomics								
MTBLS218 [37]	2016	46.8 ± 11.3	43.9 ± 5.4	WB	Pre; Post (> 1, 12 m)	44	RYGB	UHPLC–Q Exactive MS
MTBLS242 [64]	2015	Mixed	Mixed	WB	Pre; Post (3, 6, 9, 12 m)	106 + 19	RYGB; SG	NMR
MTBLS2472 [33]	2021	Mixed	Mixed	WB	Pre; Post (1 m)	25; 15	RYGB; SG	UHPLC–Orbitrap MS
MTBLS2343 [36]	2021	45.0 ± 13.0	32.21 ± 3.84	WB	Pre; Post (1, 3, 6, 12 m)	38	RYGB	UPLC–QTOF MS
PRJEB48942 [65]	2022	N/A	N/A	Stool; WB	Pre; Post (1, 3, 6, 12 m)	52	RYGB; SG	UPLC–TQ MS

*Abbreviations: AGB* adjustable gastric banding, *VBG* vertical banded gastroplasty, *SG* sleeve gastrectomy, *RYGB* Roux-en-Y gastric bypass, *WAT* white adipose tissue, *SM* skeletal muscle, *GIT* gastrointestinal tract,*PMN* peripheral mononuclear cells, *SC* subcutaneous, *AT* adipose tissue, *GM* gastric mucosa, *IE* intestinal enterocytes, *Jj* jejunum,*m* months, *w* weeks

### Transcriptomic Datasets

A total of 20 independent transcriptomic datasets were identified (Table 1). Differential expression analysis was performed using GEO2R (limma framework), comparing post- with pre-MBS samples. DEGs were defined as ≥ 1.5-fold change with FDR < 0.05; when none met this threshold, a nominal p-value < 0.05 was applied. GSE87131 yielded no DEGs at *p* < 0.05 and was excluded from downstream analysis.

Surgery type was not included as a covariate in the cross-dataset analysis because surgery-specific information was not consistently available across datasets. However, GSE271700 provided patient-level annotations by surgery type, allowing separate differential expression analyses for sleeve gastrectomy (SG) and Roux-en-Y gastric bypass (RYGB) using the same analytical approach.

Following DE analysis, DEGs were imported into Ingenuity Pathway Analysis (IPA, Qiagen) for functional analysis. IPA Core Analyses were conducted using default parameters to generate canonical pathways (CP), upstream regulators (UR), and disease/function annotations. IPA upstream regulator analysis uses the observed differential-expression patterns of known downstream targets to predict potential upstream regulators; these predictions represent inferred regulatory relationships rather than direct measurements of regulator activity.

To account for tissue-specific variation, comparative analyses were stratified by tissue type, and IPA core analysis were exported. To identify consistently regulated CPs and URs across datasets, Stouffer’s method was used to integrate activation z-scores, with separate analyses performed for CP and UR.

Two-sided p-values were computed from the combined z-scores using the standard normal distribution, and multiple-testing correction was performed using the Benjamini–Hochberg FDR method. Directional consistency was evaluated by calculating the proportion of datasets showing concordant activation or inhibition. CP and UR were considered consistently regulated when ≥ 70% of the datasets showed the same direction of predicted activity. Forest plots were generated to visualize the combined effects.

Core genes were defined as genes shared across all datasets within each tissue set and identified using Venn diagrams. Gene-level meta-analysis was performed on core genes using log2FC as the effect size. A random-effects model (REML) was applied to account for heterogeneity. Pooled estimates, 95% confidence intervals, and heterogeneity metrics (I² and τ²) were calculated. Multiple testing correction was performed using the Benjamini–Hochberg FDR method. Statistical analyses were performed in R (v4.4.3) using *dplyr*, *tidyr*, *ggplot2*, and *metafor*.

### Proteomic Datasets

Six proteomic dataset-level comparisons met the initial eligibility criteria. Two datasets (PXD063904 and PXD061410) yielded no DEPs meeting the predefined criteria and were excluded. The retained data were derived from PXD057848 and PXD065508, encompassing plasma and multiple stomach tissue comparisons (Table 1). The retained datasets, PXD057848 and PXD065508, comprised plasma (pre-MBS and 9 months post-MBS) and stomach tissues (corpus, fundus, and antrum; pre-MBS and 6 months post-MBS), respectively, analyzed by LC–MS/MS. For PXD057848, the analysis was performed using processed data, whereas for PXD065508, DEP lists were obtained from the published supplementary materials [19]. Protein-level results were subjected to the same IPA workflow described above, and CPs and URs outputs were exported for cross-dataset comparison.

Conserved UR across the four tissue/fluid types were identified by comparing IPA outputs and visualized using the *VennDiagram* package. To identify conserved UR across the proteomic datasets, IPA outputs were compared across the four tissue/fluid types. UR with activation z-scores available in all four were retained and restricted to protein symbols, excluding pharmacological and endogenous compounds. Activation z-scores for the resulting conserved regulators were visualized as a heatmap using the *pheatmap* package in R.

For CPs analysis, results from corpus, antrum, and plasma were compiled into a single matrix, with pathways below the IPA significance threshold recorded as missing. Pathway activation z-scores were visualized as a heatmap using *pheatmap*, with columns grouped by dataset accession. Due to the limited number and heterogeneity of proteomic datasets, results were pooled across tissue types to identify overall MBS-associated trends.

### Metabolomic Datasets

The included studies, analytical platforms, follow-up durations, and methodological details are summarized in Table 1. DEM identification followed the cutoff criteria applied in each respective study, with the criteria and number of DEMs identified in each dataset summarized in Table 2.

**Table 2:** Summary of differentially expressed genes, proteins, and metabolites across all included datasets and their corresponding cut-off criteria

Dataset identifier	Narrative review/Analysis	Post vs. Pre (m)	Total features	Up-regulated	Down-regulated	Cut-off (*P*-value/FDR)	
Transcriptomics							
GSE199063	Re-analyzed	24	1792	552	1240	*p* < 0.05; FDR < 0.05	
GSE199063	Re-analyzed	60	1961	563	1398	*p* < 0.05; FDR < 0.05	
GSE53376	Re-analyzed	24	563	148	415	*p* < 0.05; FDR < 0.05	
GSE65540	Re-analyzed	3	256	41	215	*p* < 0.05	
GSE59034	Re-analyzed	24	840	182	658	*p* < 0.05; FDR < 0.05	
GSE66921	Re-analyzed	24	7447	2991	4456	*p* < 0.05; FDR < 0.05	
GSE29411	Re-analyzed	4	380	112	268	*p* < 0.05	
GSE29411	Re-analyzed	12	594	167	427	*p* < 0.05	
GSE72158	Re-analyzed	12	201	49	152	*p* < 0.05; FDR < 0.05	
GSE137631	Re-analyzed	9	168	78	90	*p* < 0.05	
GSE272133	Re-analyzed	12	192	117	75	*p* < 0.05; FDR < 0.05	
GSE5109	Re-analyzed	12	1890	1238	652	*p* < 0.05	
GSE76762	Re-analyzed	3	171	124	47	*p* < 0.05	
GSE214758	Re-analyzed	6–9	1491	494	997	*p* < 0.05	
GSE113819	Re-analyzed	1	1306	515	791	*p* < 0.05	
GSE83223	Re-analyzed	6	1226	562	664	*p* < 0.05	
GSE32575	Re-analyzed	3	441	376	65	*p* < 0.05; FDR < 0.05	
GSE271700	Re-analyzed	12	1076	797	279	*p* < 0.05	
GSE271700	Re-analyzed	2	19	10	9	*p* < 0.05	
GSE19790	Re-analyzed	12	8	0	8	*p* < 0.05	
GSE66306	Re-analyzed	3	15	10	5	*p* < 0.05	
GSE126803	Re-analyzed	25% WR	198	107	91	*p* < 0.05	
Proteomics							
PXD065508*	Re-analyzed	8	204	149	55	*p* ≤ 0.05	
PXD065508**	Re-analyzed	8	43	10	33	*p* ≤ 0.05	
PXD065508***	Re-analyzed	8	374	210	164	*p* ≤ 0.05	
PXD057848	Re-analyzed	9	198	141	57	*p* ≤ 0.05	
PXD063904	Published Results	24	N/A	N/A	N/A	*p* ≤ 0.05	
PXD061410	Published Results	6	9	6	3	*p* ≤ 0.05	
PXD061410	Published Results	12	N/A	N/A	N/A	*p* ≤ 0.05	
Metabolomics							
MTBLS218	Published Results	12	177	26	31	*p* < 0.001	
MTBLS242	Published Results	12	30	22	15	*p* < 0.005	
MTBLS2472	Published Results	1	61	6	20	*p* < 0.001	
MTBLS2343	Published Results	12	27	5	N/A	*p* < 0.05	
PRJEB48942	Published Results	12	38	4	10	*p* < 0.05, 0.01, 0.001	

*Abbreviations: m* Months, *WR* Weight Reduction.*Corpus tissue; **Fundus tissue; ***Antrum tissue

The extracted metabolites were manually grouped into major metabolite categories using Kyoto Encyclopedia of Genes and Genomes (KEGG)-based annotation, including amino acids, lipids, bile acids, central carbon and energy metabolites, microbial-derived metabolites, and other small-molecule classes. This classification was used to summarize the distribution of altered metabolites across studies and to identify metabolic classes commonly affected following MBS (Fig. 4). Due to the limited number ofdatasets, differences in analytical platforms and the lack of uniformly processed quantitative data across studies, formal pathway- or metabolite-level meta-analysis was not performed the findings wereanalyzed descriptively across studies.

## Results

The transcriptomic, proteomic, and metabolomic datasets spanning 2006–2026, with diverse cohorts of adults with obesity (mean age ~ 35–50 years; BMI ~ 40–50 kg/m²) were included in this study (Table 1). Sample sizes and types varied substantially across studies (*n* = 6–165), with some datasets lacking complete demographic informationPostoperative time points ranged from early (1 week – 3 months) to long-term follow-up (up to 60 months). RYGB was the most commonly investigated surgical procedure across the datasets, although sleeve gastrectomy, adjustable gastric banding, and other techniques were also represented (Table 1). The total numbers of DEGs, DEPs and DEMs for each dataset are presented in Table 2.

### Transcriptomics 

**Fig. 1:**
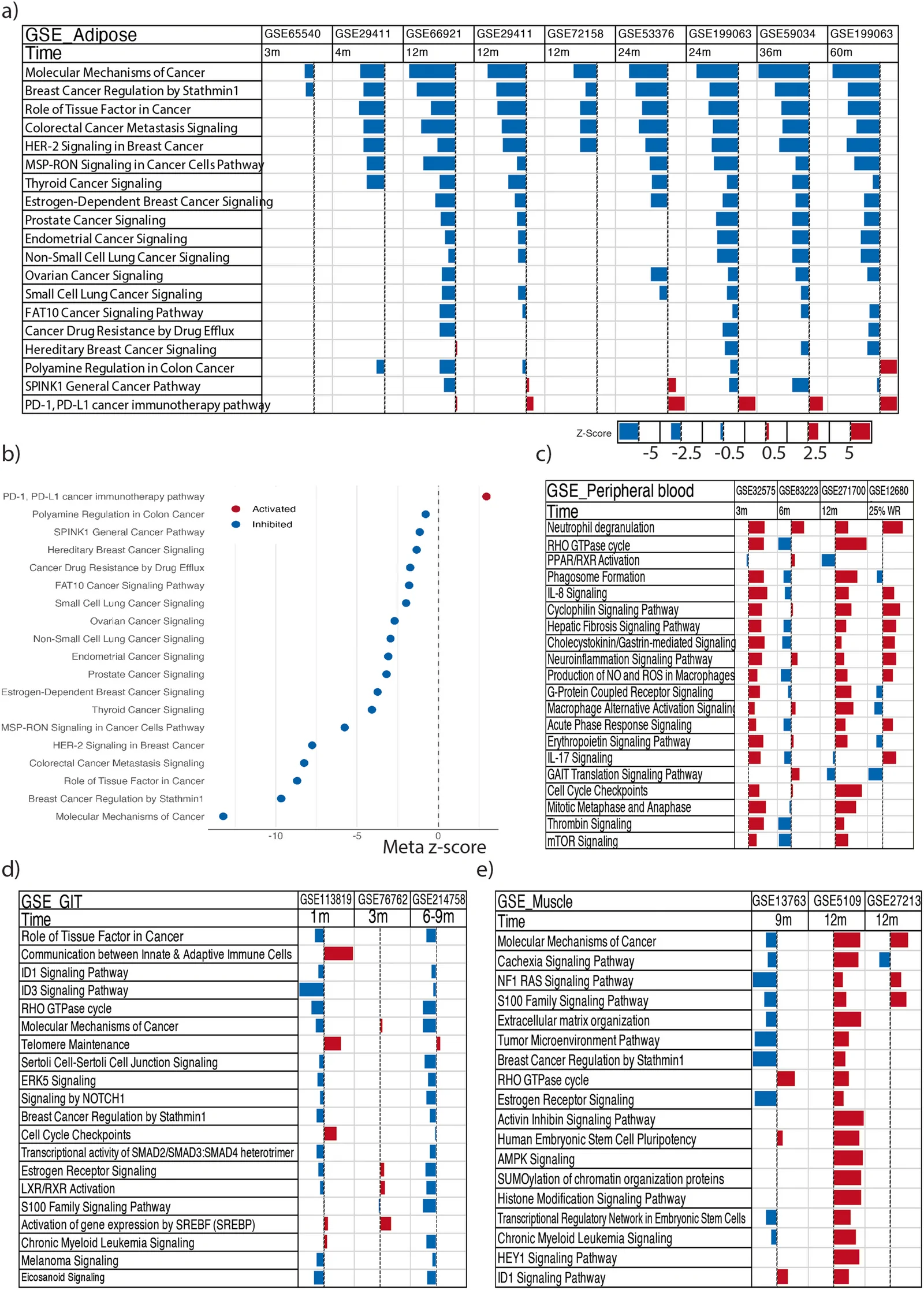
IPA-generated canonical pathways regulation across all transcriptomic datasets. Differentially expressed genes identified from each dataset were analyzed using IPA to determine the predicted functional activity of established canonical pathways. Pathway activity was inferred using the IPA activation Z-score, where positive Z-scores (red in heatmaps) indicate predicted pathway activation and negative Z-scores (blue in heatmaps) indicate predicted pathway inhibition. The results are stratified by tissue type: (**a**, **b**) adipose tissue, (**c**) peripheral blood, (**d**) gastrointestinal tract (GIT), and (**e**) skeletal muscle datasets

IPA predicted consistent inhibition of cancer-related pathways across adipose tissue datasets, with stronger inhibition in several datasets with longer follow-up durations (12–60 months). Sustained inhibition was observed for *Molecular Mechanisms of Cancer*, *Breast Cancer Regulation by Stathmin1*, *Role of Tissue Factor in Cancer*, *Colorectal Cancer Metastasis Signaling*, and *HER-2 Signaling* (Fig. 1a, b). In contrast, the *PD-1/PD-L1 Cancer Immunotherapy Pathway* showed increased activity across datasets (Fig. 1a, b).

Peripheral blood showed predominant activation of immune and inflammatory pathways post-surgery, including *Neutrophil Degranulation*,* Phagosome Formation*,* IL-8 Signaling*,* Neuroinflammation Signaling*,* ROS Production in Macrophages*, and *Acute Phase Response* (Fig. 1c). *Cell Cycle Checkpoints* and mitotic pathways were also activated in GSE32575 and GSE271700. GIT datasets showed patterns similar to adipose tissue, with sustained inhibition of *Molecular Mechanisms of Cancer*,* RHO GTPase Cycle*,* NOTCH1 Signaling*,* ERK5 Signaling*, and *Estrogen Receptor Signaling* (Fig. 1d). Peripheral blood and skeletal muscle datasets showed greater variability in pathway regulation than adipose and GIT datasets (Fig. 1c and e).

**Fig. 2:**
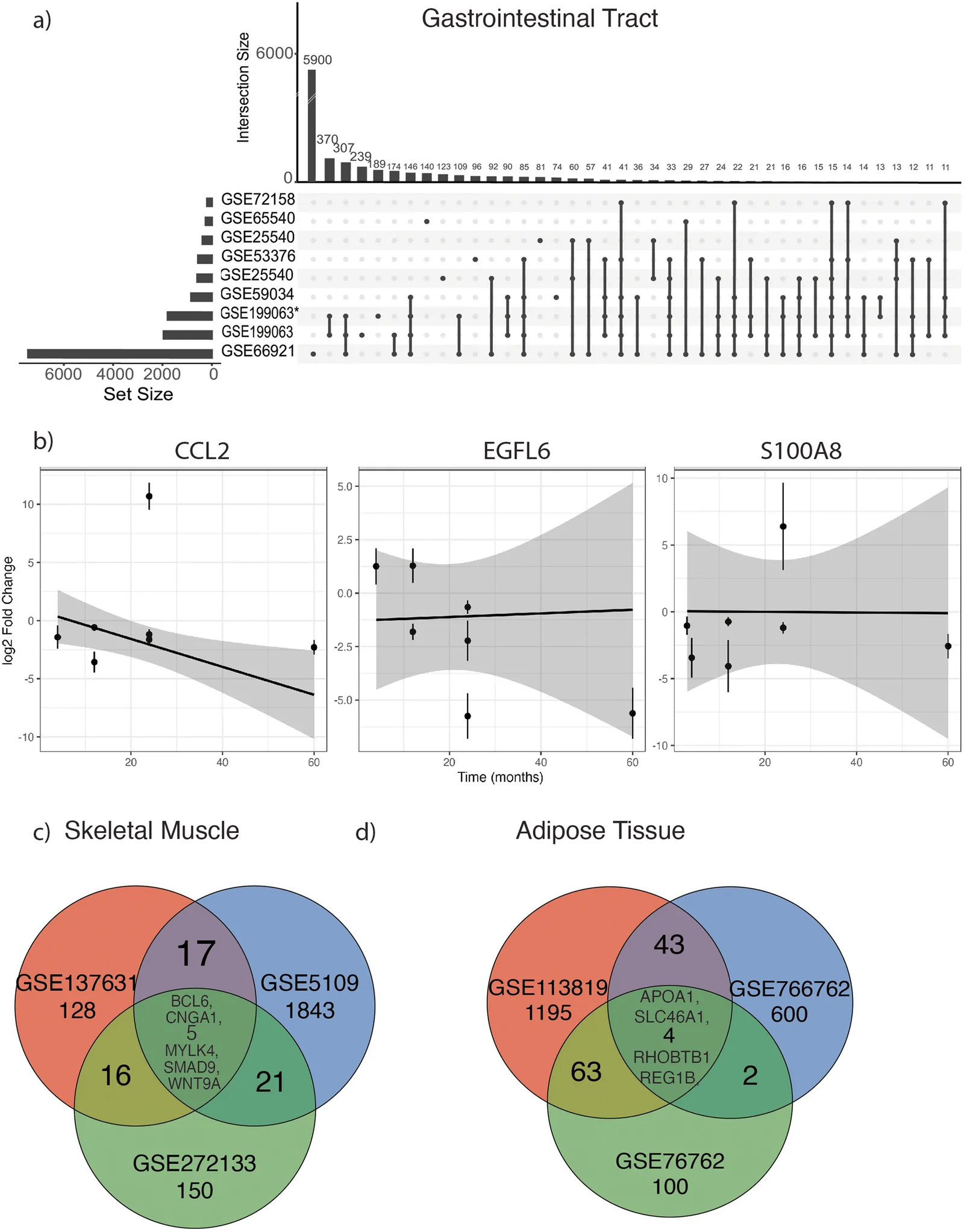
Shared (core) genes across transcriptomic datasets identified by REML-based meta-analysis. (**a**) UpSet plot showing the overlap of DEGs across adipose tissue datasets. (**b**) Weighted meta-regression analyses of representative core adipose tissue genes (CCL2, EGFL6, and S100A8), illustrating the relationship between log2FC and disease duration (months) in adipose tissue datsets. Bubble size is proportional to study weight, solid lines represent weighted linear regression, and shaded areas indicate 95% confidence intervals. (**c**, **d**) Venn diagram showing shared DEGs among skeletal muscle (**c**) and GIT datasets (**d**)

UpSet analysis identified genes shared across multiple adipose tissue cohorts (Fig. 2a). Among the most frequently shared DEGs, C-C motif chemokine ligand 2 (*CCL2*), epidermal growth factor-like protein 6 (*EGFL6*), and S100 calcium-binding protein A8 (*S100A8*) were each identified in approximately 80% of adipose tissue datasets. *CCL2* and *S100A8* were downregulated in all but one dataset, whereas *EGFL6* was downregulated in all but two datasets (Fig. 2b). Skeletal muscle datasets identified five core genes: B-cell lymphoma 6 (*BCL6*), cyclic nucleotide-gated channel alpha 1 (*CNGA1*), myosin light chain kinase family member 4 (*MYLK4*), SMAD family member 9 (*SMAD9*), and Wnt family member 9 A (*WNT9A*) (Fig. 2c; Supplementary Fig. 4a). GIT datasets identified four core genes: apolipoprotein A1 (*APOA1*), regenerating family member 1 beta (*REG1B*), Rho-related BTB domain containing 1 (*RHOBTB1*), and solute carrier family 46 member 1 (*SLC46A1*) (Fig. 2d; Supplementary Fig. 4b).

UR analysis revealed distinct tissue-specific regulatory patterns (Supplementary Fig. 5). In adipose tissue (Supplementary Fig. 5a), most UR were predicted to be inhibited, including inflammatory cytokines (TNF, IL1B, IL6, and IFNG), signaling mediators such as RELA, STAT3, and TGFB1, and components of the PI3K pathway. In skeletal muscle (Supplementary Fig. 5b), metabolic regulators, including ADIPOQ, PPARG, SREBF1, and PTEN, were predicted to be inhibited, whereas signaling and growth-related regulators, including AKT1, TP53, TGFB1, STAT1, ERK, and WNT3A, were predicted to be activated. In GIT tissue (Supplementary Fig. 5c), inflammatory cytokines (IL1B, IFNG, and IL6) and regulatory factors, including PI3K, AKT, STAT1, RELA, VEGFA, and CTNNB1, were predicted to be activated, whereas TGFB1, TP53, and MAPK14 were predicted to be inhibited.

We further examined whether these changes differed by MBS type. Although both SG and RYGB were represented across the five transcriptomic datasets, only GSE271700 provided sufficient patient-level annotation for surgery-specific differential expression analysis. Almost all DEGs were identified following SG, compared with only four following RYGB, indicating that the transcriptomic changes in this dataset were predominantly driven by the SG group. Overall, the available data were insufficient for a robust comparison of transcriptomic remodeling between SG and RYGB across datasets.

### Proteomics 

**Fig. 3:**
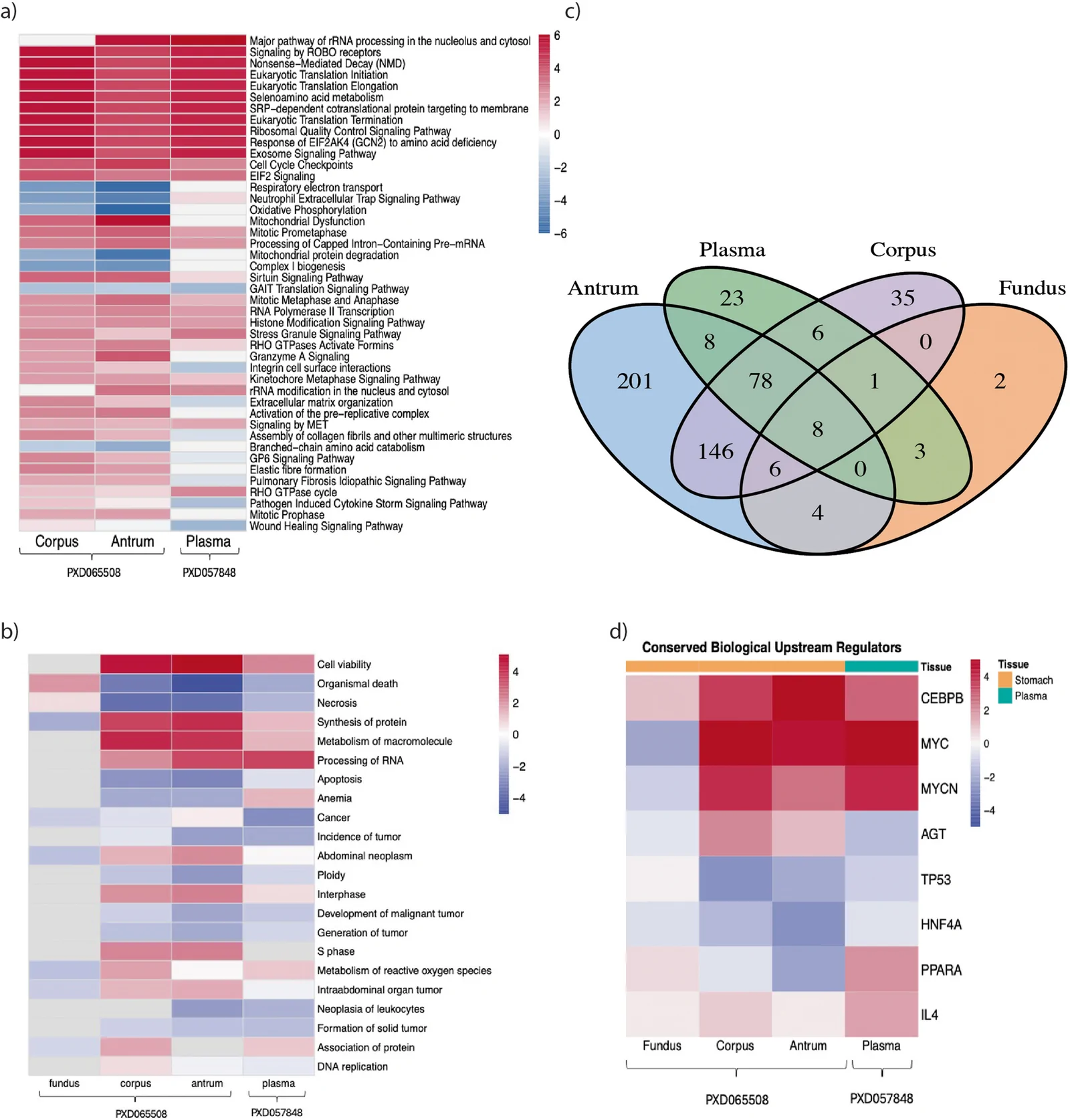
IPA analysis of proteomics datasets across plasma and gastrointestinal tissues. (**a**) Heatmap of canonical pathways enriched in corpus, antrum, and plasma, ranked by Z-score; pathway-level data were not available for fundus. (**b**) Heatmap of diseases and biological functions across fundus, corpus, antrum, and plasma, based on IPA-predicted activation (positive Z-score) or inhibition (negative Z-score). (**c**) Venn diagram showing the overlap of proteins identified by mass spectrometry across antrum, corpus, fundus, and plasma (PXD065508, PXD057848). (**d**) Heatmap of upstream regulators conserved across all four tissues (fundus, corpus, antrum, plasma), based on IPA-predicted activation Z-scores

CP analysis demonstrated consistent predicted activation of pathways such as *EIF2 Signaling* and *Cell Cycle Checkpoints* across corpus, antrum, and plasma datasets (Fig. 3a). In contrast, the *GAIT Translation Signaling Pathway* was consistently predicted to be inhibited following MBS. Functional analysis of diseases and biological functions revealed several key processes altered following MBS (Fig. 3b). Cancer-related functions were predominantly predicted to be inhibited after surgery. Although not all samples showed uniform patterns, processes such as apoptosis, tumor formation, and ploidy were consistently predicted to be inhibited across three datasets. Conversely, RNA processing and cell viability were predicted to be activated in corpus, plasma, and antrum samples. The fundus dataset showed the fewest significantly enriched biofunctions.

Comparative UR analysis identified shared regulators across all comparisons (Fig. 3c). The Venn diagram identified eight consistently detected UR, including predicted activation of *CEBPB* and *IL4* and inhibition of *TP53* and *HNF4A* (Fig. 3c, d). Additional regulators, including *MYC* and *MYCN*, were selectively predicted to be activated in plasma, corpus, and antrum samples (Fig. 3d).

### Metabolomics 

**Fig. 4:**
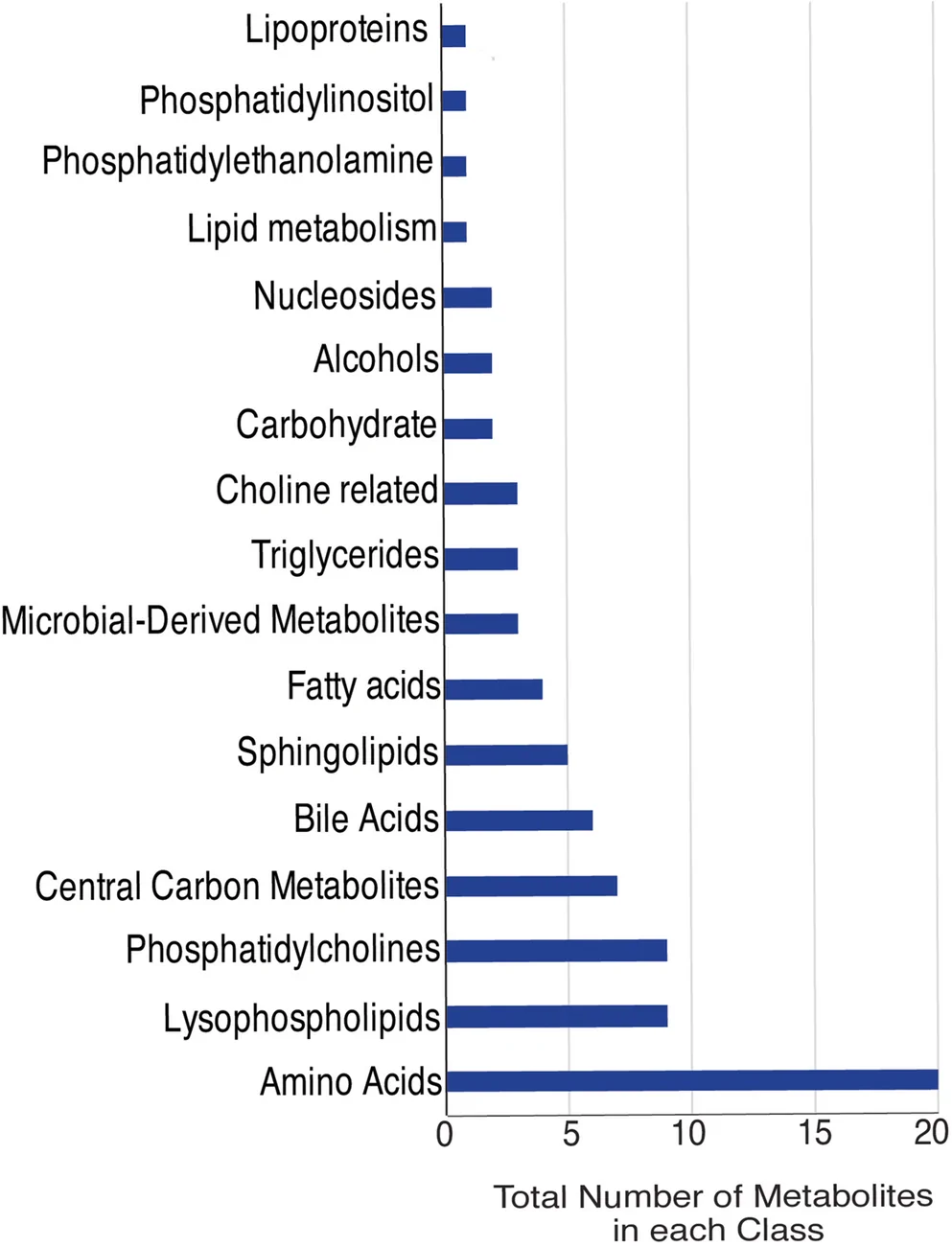
Classification of differentially expressed metabolites identified across all studies into major metabolite classes based on KEGG annotation

Metabolomic analysis identified diverse classes of metabolites following MBS (Fig. 4). Most DEMs were amino acids (*n* = 20), followed by lysophospholipids (*n* = 9), phosphatidylcholines (*n* = 9), energy and central carbon metabolites (*n* = 7), bile acids (*n* = 6), sphingolipids (*n* = 5), and fatty acids (*n* = 4) (Fig. 4).

Metabolite changes varied by dataset and postoperative time point (Table 3). MTBLS218 showed early increases in TMAO, acetylglycine, arginine, carnitine, glycine, glutamine, and phospholipid species, with several metabolites decreasing at later time points.(Table 3, top panel). In MTBLS242, amino acids and central carbon metabolites were induced in severe obesity compared to controls, whereas post-surgery profiles demonstrated upregulation of arginine, glutamine, dimethyl sulfone, trimethylamine N-oxide (TMAO), and formate, alongside downregulation of BCAAs and aromatic amino acids, as well as citrate and pyruvate (Table 3).

**Table 3:** Upregulated and downregulated metabolites across all metabolomics datasets included in this study (post-MBS vs. pre-MBS)

Datasets		MTBLS218 Post MBS Vs Pre MBS			MTBLS242			MTBLS2472	MTBLS2343	PRJEB48942
		0–3 weeks	1–3 weeks	12 months	Severe Obesity vs. Cont		Post-MBS vs. Pre MBS	1 month	12 months	12 months
Differentially Expressed Metabolties	Upregulated	Trimethylamine-N-oxide	Acetylglycine	Creatine	Alanine		Arginine	p-Cresol sulfate	Hyocholic acid	Dimethyl sulfone
		Phosphatidylcholine C40:7	Arginine	LysoPC C16:1	Phenylalanine		Glutamine	Acetyl-carnitine	Hyodeoxycholic acid	Glycine
		Indoxyl sulfate	Carnitine	LysoPC C18:2	Tyrosine		Dimethyl sulfone	Decanoylcarnitine	Glycohyocholic acid	O-acetylcholine
		Hydroxydecanoic acid	Hydroxyisobutyric acid	Ornithine	Leucine		TMAO	Sphingomyelin (d36:1)	Glycohyodeoxycholic acid	Glutamine
		Glycine	Leu-Pro (dipeptide)	PC C34:3	Isoleucine		Formate	Sphingomyelin (d36:2)	Total HCA species	
		Glutamine	LysoPE C20:4	PC C36:5	Valine		LDL1	Glycochenodeoxycholic acid		
			Pantothenic acid	PC C36:6	Citrate		Glycine			
			PC C38:6	Sarcosine	Formate		Histidine			
			Pyroglutamic acid	Tryptophan	Pyruvate					
			Threonine	Uracil	Acetoacetate (trend)					
					Glucose					
					Isopropanol					
					Methanol					
					VLDL1					
	Down reguleted	Creatine	Acetylglycine	Alanine	Glutamine		Tyrosine	LPC (14:0, 15:0, 16:1, 18:0, 18:3, 20:5, 22:5)	Not Reported	Isoleucine
		LysoPC (C16:1, C18:2)	Arginine	Choline	Histidine		Phenylalanine			Tyrosine
		Ornithine	Carnitine	Leucine/Isoleucine			Tyrosine	PC (32:1, 34:3, 36:3, 38:3, 38:7)		Valine
		PC (C34:3, C36:5, C36:6)	Hydroxyisobutyric acid	Lysine			Pyruvate	PE (34:2)		Alanine
		Sarcosine	Leu-Pro (dipeptide)	Oxovaleric acid			Citrate (transient)	PI (38:3)		Glucose
		Tryptophan	LysoPE (C20:4)	Pentoses			Acetoacetate/3-HB	SM (d40:1, d41:1, d42:1)		Glutamate
		Uracil	Pantothenic acid	Uridine			Isopropano	TG (48:1, 50:3, 50:4)		Lactate
			PC (C38:6)	Valine			Methano	VLDL1		Mannose
			Pyroglutamic acid	Tyrosine			Formate			Glycerol
			Threonine	Phenylalanine*			Leucine			Creatine
							Isoleucine			Leucine
							Valine			

*3-HB* 3-hydroxybutyrate, *HCA* hyocholic acid, *LDL* low-density lipoprotein, *LPC* lysophosphatidylcholine, *LysoPC* lysophosphatidylcholine, *LysoPE* lysophosphatidylethanolamine, *MBS* metabolic and bariatric surgery, *PC* phosphatidylcholine, *PE* phosphatidylethanolamine, *PI* phosphatidylinositol, *SM* sphingomyelin, *TG* triglyceride, *TMAO* trimethylamine N-oxide, *VLDL* very-low-density lipoprotein

MTBLS2472 showed upregulation of microbial-derived and lipid metabolites, including p-cresol sulfate, acyl-carnitines, sphingomyelins, and bile acid derivatives, with concurrent downregulation of multiple lipid classes, including phosphatidylcholines, phosphatidylethanolamines, phosphatidylinositols, sphingomyelins, triglycerides, and lipoproteins. MTBLS2343 demonstrated increased levels of bile acid species at 12 months, while PRJEB48942 identified upregulation of dimethyl sulfone, glycine, O-acetylcholine, and glutamine, and downregulation of amino acids and energy-related metabolites, including leucine, isoleucine, valine, glucose, lactate, and glycerol (Table 3). Across datasets increased levels of amino acids and microbial-associated metabolites and dercease downregulated metabolites included BCAAs and lipid species, particularly phosphatidylcholines and related lipid class es.

## Discussion

This multi-omics study provides an overview of molecular changes associated with MBS. Overall, the findings indicate tissue-specific alterations in cancer-related, immune, and metabolic pathways following surgery. While epidemiological studies have reported reduced overall and site-specific cancer risk following MBS in individuals with obesity [20], the present study provides insight into the molecular changes occurring after MBS that may be relevant to these epidemiological observations. However, heterogeneity in patient characteristics, surgical procedures, tissues, and follow-up periods should be considered when interpreting cross-dataset patterns.

Predicted inhibition of several cancer-associated CPs was observed consistently in adipose and GIT datasets. Some adipose tissue datasets with longer postoperative follow-up showed greater predicted inhibition of these pathways, potentially reflecting sustained molecular changes over time following MBS. Increased predicted activity of the PD-1/PD-L1 Cancer Immunotherapy Pathway was also observed, consistent with a previous pilot study, although the functional implications of this finding require further investigation [21]. However, these pathway-level predictions should not be interpreted as direct evidence of reduced tumorigenic activity or cancer risk, as cancer incidence following MBS was not assessed in this study.

The predominant downregulation of *CCL2* and *S100A8* may be relevant to the modulation of obesity-associated inflammation following MBS [22–25]. Notably, Urinary CCL2 has been suggested as a promising biomarker for assessing improvement in renal function in individuals with obesity following MBS [25]. *EGFL6*, associated with adipose dysfunction and angiogenesis [26, 27], was similarly downregulated in most datasets. Collectively, these findings suggest that MBS-associated weight loss is accompanied by sustained transcriptional remodeling of inflammatory and angiogenic processes in adipose tissue.

Proteomic analysis also predicted inhibition of several cancer-related biological functions and GAIT translation while EIF2 signaling and predicted activation of cell-cycle checkpoint pathways suggesting changes in protein synthesis, cellular stress responses, and cell-cycle regulation [28]. UR analysis further identified shared regulatory patterns across plasma and GIT tissues, including predicted activation of CEBPB and IL4 and inhibition of TP53 and HNF4A, consistent with broader changes in immune and metabolic regulatory networks. However, the biological implication of these pathway predictions cannot be established from the present analysis.

Metabolomic analysis revealed postoperative alterations across amino acid, lipid, bile acid, and energy-related metabolite classes. BCAA levels, including leucine, isoleucine, and valine, were reduced across multiple datasets. Elevated circulating BCAAs have been associated with obesity and insulin resistance [29, 30], while reductions following MBS have been linked to metabolic improvement. Notably, proteomic analysis also identified alterations in BCAA catabolism in corpus and antrum tissues (Fig. 3a), suggesting BCAA-related remodeling at both the proteomic and metabolomic levels following MBS.

Alterations in lipid metabolism involved phosphatidylcholines, carnitines, sphingolipids, triglycerides, and related lipid classes, consistent with previous studies reporting changes in circulating lipid profiles [31, 32] and liver function following MBS [33]. Bile acids and microbiome-associated metabolites were also altered. Post-MBS bile acid profiles have been linked to changes in enterohepatic signaling through pathways including FXR and TGR5 [34–36], while metabolites such as TMAO, dimethyl sulfone, and p-cresol sulfate may reflect alterations in gut microbial metabolism [37]. However, individual lipid species varied across studies and postoperative time points, and microbiome composition and receptor signaling were not directly assessed. Therefore, these metabolomic associations do not establish specific microbiome-mediated mechanisms. However, individual lipid species varied across studies and postoperative time points, and microbiome composition and receptor signaling were not directly assessed. Therefore, these metabolomic associations do not establish specific microbiome-mediated mechanisms.

Proteomic analysis also identified pathways related to mitochondrial function, oxidative phosphorylation, and complex I biogenesis. In parallel, metabolomic datasets showed postoperative changes in central carbon metabolites, including pyruvate, lactate, citrate, and formate. These observations indicate changes in energy-related processes across the proteomic and metabolomic datasets; however, their functional significance cannot be determined from the present analyses.

An important consideration when interpreting these findings is the relative contribution of weight loss and the surgical intervention itself. Although not investigated in this study, weight loss is likely to contribute substantially to the metabolic and inflammatory changes observed following MBS. At the same time, MBS is associated with anatomical and physiological changes, including alterations in gut hormone secretion, bile acid metabolism, and enterohepatic signaling [34, 35, 38]. These mechanisms may contribute to the molecular patterns observed after surgery; however, the datasets included in the present study did not contain non-surgical weight-loss comparator groups. Therefore, the relative contributions of surgery-specific mechanisms and weight loss cannot be determined from the current analysis.

**Fig. 5:**
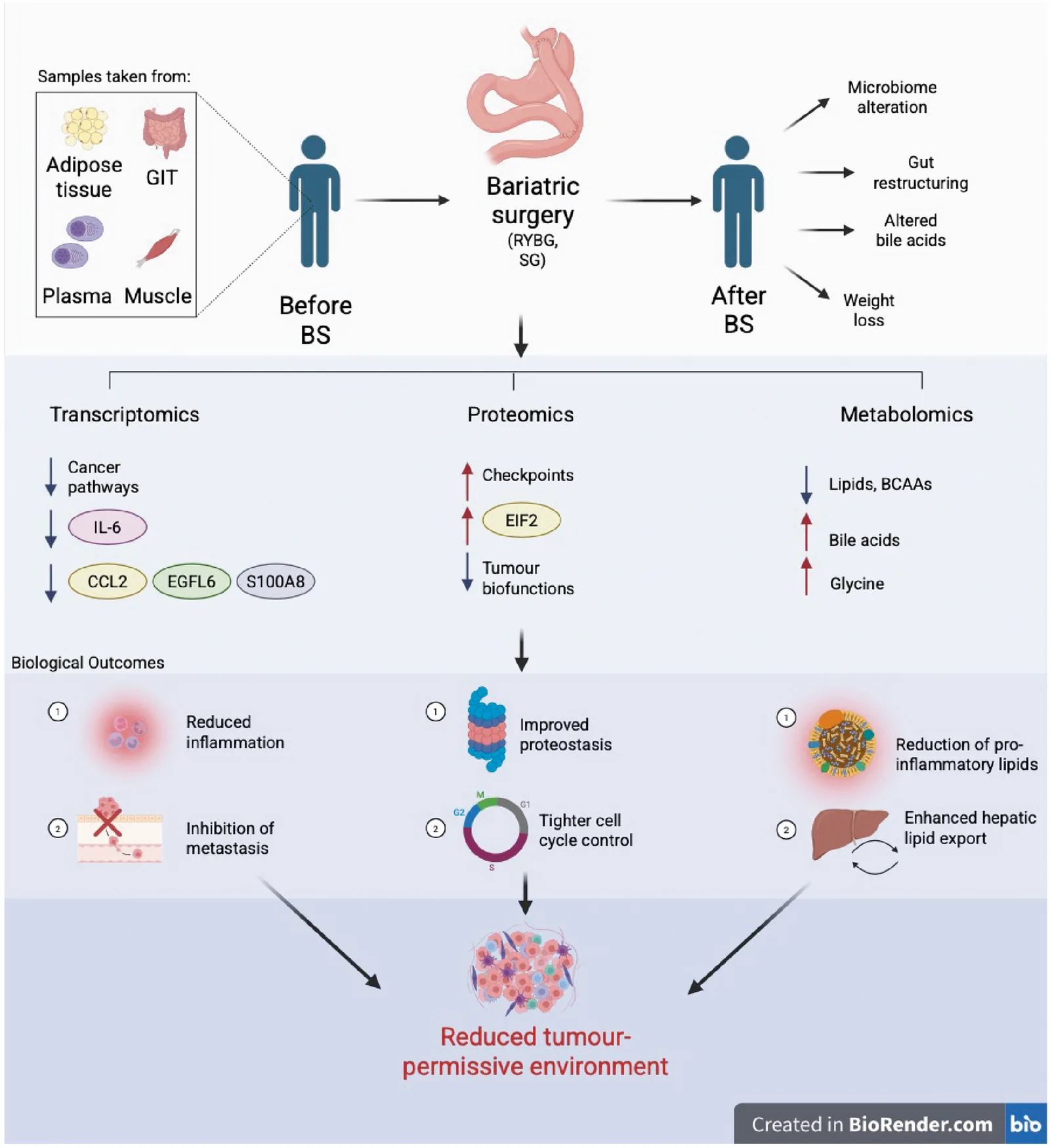
Proposed overview of molecular changes associated with metabolic bariatric surgery (MBS) across transcriptomic, proteomic, and metabolomic datasets. Samples collected before and after MBS were evaluated across multiple tissues and biological samples. Transcriptomic findings included changes in cancer-related and inflammatory pathways and genes, including *IL6*, *CCL2*, *EGFL6*, and *S100A8*. Proteomic findings indicated alterations in EIF2 signaling, cell-cycle checkpoints, and tumor-related biological functions, while metabolomic findings showed changes in lipids, branched-chain amino acids (BCAAs), bile acids, and glycine. The proposed biological outcomes shown represent an integrated interpretation of the molecular patterns identified across the omics datasets. Upward and downward arrows indicate increased and decreased abundance or predicted pathway/function activity, respectively

Overall, the three omics layers showed changes associated with MBS across several biological processes (Fig. 5), including cancer-associated and immune pathways, protein synthesis and cell-cycle-related pathways, and amino acid, lipid, and bile acid metabolism. These findings provide a descriptive multi-omics overview of molecular changes following MBS and identify patterns for further investigation.

### Study Limitations

A key limitation is the use of nominal *p* < 0.05 thresholds for datasets in which no genes met the predefined FDR threshold, potentially increasing false-positive findings. IPA predictions represent inferred regulatory trends rather than direct functional measurements, and the lack of epidemiological and patient-level outcomes precludes causal inference regarding cancer risk or clinical relevance. IPA anlaysis are also dependent on the composition and coverage of the underlying knowledge base and may be influenced by literature and sampling biases, with extensively studied genes and pathways potentially being more strongly represented. Furthermore, without non-surgical weight-loss comparators, MBS-associated molecular changes cannot be distinguished from those attributable to weight loss itself. Heterogeneity in surgical procedures, patient characteristics, follow-up periods, sample types, and analytical platforms further limits cross-study comparisons. These findings should therefore be considered hypothesis-generating and require validation in prospective longitudinal and experimental studies with appropriate non-surgical weight-loss comparators.

## Conclusion

This multi-omics analysis identified molecular changes across transcriptomic, proteomic, and metabolomic datasets when comparing post-MBS with pre-MBS samples. The findings showed tissue-specific alterations in cancer-associated and immune pathways, protein synthesis and cell-cycle-related processes, and amino acid, lipid, bile acid, and energy metabolism. Related changes in BCAAs and energy metabolism were also observed across proteomic and metabolomic datasets, suggesting overlapping molecular patterns across omics layers. While these findings provide a broader molecular perspective on the biological changes associated with MBS, they should not be interpreted as evidence of causal mechanisms or direct cancer-protective effects. Further prospective studies with standardized sampling, appropriate non-surgical weight-loss comparators, and clinical outcome data are needed to validate these molecular patterns and determine their biological and clinical relevance.

## Supplementary Information

Below is the link to the electronic supplementary material.

Supplementary Material 1 Details of the literature search and selection of transcriptomic datasets. (JPG 1.93 MB)

Supplementary Material 2 Details of the literature search and selection of proteomic datasets. (JPG 554 KB)

Supplementary Material 3Details of the literature search and selection of metabolomic datasets. (JPG 501 KB)

Supplementary Material 4 (**a**) Weighted meta-regression analyses of representative skeletal muscle genes (BCL6, CNGA1, MYLK4, SMAD9, and WNT9A). (**b**) Weighted meta-regression analyses of representative GIT genes (APOA1, REG1B, RHOBTB1, and SLC46A1). (JPG 559 KB)

Supplementary Material 5 The panels show the meta‑analysis of upstream regulators for adipose tissue (**a**), skeletal muscle (**b**), and GIT (**c**). (JPG 1.96 MB)

## Data Availability

No datasets were generated or analysed during the current study.

## References

[1] Reilly JJ, Methven E, McDowell ZC, Hacking B, Alexander D, Stewart L, et al. Health consequences of obesity. Arch Dis Child. 2003;88(9):748–52. 10.1136/adc.88.9.748. PMC1719633 12937090

[2] Dobbins M, Decorby K, Choi BC. The Association between Obesity and Cancer Risk: A Meta-Analysis of Observational Studies from 1985 to 2011. ISRN Prev Med. 2013;2013:680536. 10.5402/2013/680536. PMC4062857 24977095

[3] Liu X, Ju W, Huo C, Zhang S, Wang X, Huang K. Overweight and obesity as independent factors for increased risk of hepatocellular cancer-related mortality: a meta-analysis. J Am Coll Nutr. 2021;40(3):287–93. 10.1080/07315724.2020.1751007. 32281914

[4] O’Sullivan DE, Sutherland RL, Town S, Chow K, Fan J, Forbes N, et al. Risk Factors for Early-Onset Colorectal Cancer: A Systematic Review and Meta-analysis. Clin Gastroenterol Hepatol. 2022;20(6):1229–e405. 10.1016/j.cgh.2021.01.037. 33524598

[5] Lu Y, Tao J. Diabetes mellitus and obesity as risk factors for bladder cancer prognosis: a systematic review and meta-analysis. Front Endocrinol (Lausanne). 2021;12:699732. 10.3389/fendo.2021.699732. PMC8529220 34690923

[6] Chlebowski RT, Aragaki AK, Pan K, Simon MS, Neuhouser ML, Haque R, et al. Breast cancer incidence and mortality by metabolic syndrome and obesity: the Women’s Health Initiative. Cancer. 2024;130(18):3147–56. 10.1002/cncr.35318. 38736319

[7] Guh DP, Zhang W, Bansback N, Amarsi Z, Birmingham CL, Anis AH. The incidence of co-morbidities related to obesity and overweight: a systematic review and meta-analysis. BMC Public Health. 2009;9:88. 10.1186/1471-2458-9-88. PMC2667420 19320986

[8] Lin Y, Wang Y, Wu Q, Jin H, Ma G, Liu H, et al. Association between obesity and bladder cancer recurrence: a meta-analysis. Clin Chim Acta. 2018;480:41–6. 10.1016/j.cca.2018.01.039. 29408169

[9] Brinton LA, Trabert B, Anderson GL, Falk RT, Felix AS, Fuhrman BJ, et al. Serum Estrogens and Estrogen Metabolites and Endometrial Cancer Risk among Postmenopausal Women. Cancer Epidemiol Biomarkers Prev. 2016;25(7):1081–9. 10.1158/1055-9965.epi-16-0225. PMC4930692 27197275

[10] Leitner BP, Siebel S, Akingbesote ND, Zhang X, Perry RJ. Insulin and cancer: a tangled web. Biochem J. 2022;479(5):583–607. 10.1042/bcj20210134. PMC9022985 35244142

[11] Knuppel A, Fensom GK, Watts EL, Gunter MJ, Murphy N, Papier K, et al. Circulating Insulin-like Growth Factor-I Concentrations and Risk of 30 Cancers: Prospective Analyses in UK Biobank. Cancer Res. 2020;80(18):4014–21. 10.1158/0008-5472.can-20-1281. 32709735

[12] Maciejewski ML, Arterburn DE, Van Scoyoc L, Smith VA, Yancy WS Jr, Weidenbacher HJ, et al. Bariatric Surgery and Long-term Durability of Weight Loss. JAMA Surg. 2016;151(11):1046–55. 10.1001/jamasurg.2016.2317. PMC5112115 27579793

[13] Wilson RB, Lathigara D, Kaushal D. Systematic review and meta-analysis of the impact of bariatric surgery on future cancer risk. Int J Mol Sci. 2023. 10.3390/ijms24076192. PMC10094585 37047163

[14] Bagheri M, Tanriverdi K, Iafrati MD, Mosley JD, Freedman JE, Ferguson JF. Characterization of the plasma metabolome and lipidome in response to sleeve gastrectomy and gastric bypass surgeries reveals molecular patterns of surgical weight loss. Metabolism. 2024;158:155955. PubMed PMID: 38906372; PubMed Central PMCID: PMC11755375. 10.1016/j.metabol.2024.155955 PMC11755375 38906372

[15] Almazrouei B, Mousa M, Barreiros A, Al Dain Marzouka N, Olbrich M, Ramachandran S, et al. Multi-omics analysis of bariatric surgery’s impact on type 2 diabetes and prediabetes. Sci Rep. 2025;15(1):29215. 10.1038/s41598-025-14577-w. PMC12335437 40783429

[16] Bouchard-Mercier A, de Toro-Martín J, Nadeau M, Lescelleur O, Lebel S, Richard D, et al. Molecular remodeling of adipose tissue is associated with metabolic recovery after weight loss surgery. J Transl Med. 2022;20(1):283. 10.1186/s12967-022-03485-6. PMC9219157 35739539

[17] Lamabadusuriya DA, Jayasena H, Bopitiya AK, De Silva AD, Jayasekera P. Obesity-driven inflammation and cancer risk: A comprehensive review. Semin Cancer Biol. 2025;114:256–66. 10.1016/j.semcancer.2025.07.007. 40714142

[18] Yang M, Zhu C, Du L, Huang J, Lu J, Yang J, et al. A Metabolomic Signature of Obesity and Risk of Colorectal Cancer: Two Nested Case–Control Studies. Metabolites. 2023;13(2):234. 10.3390/metabo13020234. PMC9965372 36837854

[19] Larson CIW, Fändriks L, Casselbrant A, Wallenius V. Novel insights on remnant stomach following Roux-en-Y gastric bypass surgery based on histological evaluation and quantitative proteomics analysis. Sci Rep. 2025;15(1):25243. 10.1038/s41598-025-10114-x. PMC12255712 40652086

[20] Madar LO, Goldberg N, Netz U, Berenstain IF, Abu Zeid EED, Avital I, et al. Association between metabolic and bariatric surgery and malignancy: a systematic review, meta-analysis, trends, and conclusions. Surg Obes Relat Dis. 2025;21(4):434–48. 10.1016/j.soard.2024.10.023. 39581814

[21] Cucoreanu C, Muresan XM, Tigu AB, Nistor M, Moldovan RC, Pralea IE, et al. The Relationship between Proinflammatory Molecules and PD-L1 in Patients with Obesity Who Underwent Gastric Sleeve Surgery-A Pilot Study. Rep (MDPI). 2024;7(3). 10.3390/reports7030074. PMC12225238 40729297

[22] Lylloff L, Bathum L, Madsbad S, Grundtvig JLG, Nordgaard-Lassen I, Fenger M. S100A8/A9 (Calprotectin), Interleukin-6, and C-reactive protein in obesity and diabetes before and after Roux-en-Y gastric bypass surgery. Obes Facts. 2017;10(4):386–95. 10.1159/000478097. PMC5644965 28848164

[23] Riuzzi F, Chiappalupi S, Arcuri C, Giambanco I, Sorci G, Donato R. S100 proteins in obesity: liaisons dangereuses. Cell Mol Life Sci. 2020;77(1):129–47. 10.1007/s00018-019-03257-4. PMC11104817 31363816

[24] Weisberg SP, Hunter D, Huber R, Lemieux J, Slaymaker S, Vaddi K, et al. CCR2 modulates inflammatory and metabolic effects of high-fat feeding. J Clin Invest. 2006;116(1):115–24. 10.1172/jci24335. PMC1307559 16341265

[25] Said SB, Loo GH, Kosai NR, Rajan R, Mohd R, Wahab AA, et al. Reduction in urinary chemokine (C-C motif) ligand 2 (CCL2) after surgery-induced weight loss. Sci Rep. 2020;10(1):790. 10.1038/s41598-020-57763-8. PMC6972822 31964990

[26] Garrett AA, Bai S, Cascio S, Gupta N, Yang D, Buckanovich RJ. EGFL6 promotes endometrial cancer cell migration and proliferation. Gynecol Oncol. 2024;185:75–82. 10.1016/j.ygyno.2024.02.016. PMC11179989 38368816

[27] Kang J, Wang J, Tian J, Shi R, Jia H, Wang Y. The emerging role of EGFL6 in angiogenesis and tumor progression. Int J Med Sci. 2020;17(10):1320–6. 10.7150/ijms.45129. PMC7330666 32624687

[28] Lassailly G, Caiazzo R, Ntandja-Wandji LC, Gnemmi V, Baud G, Verkindt H, et al. Bariatric Surgery Provides Long-term Resolution of Nonalcoholic Steatohepatitis and Regression of Fibrosis. Gastroenterology. 2020;159(4):1290–e3015. 10.1053/j.gastro.2020.06.006. 32553765

[29] Lynch CJ, Adams SH. Branched-chain amino acids in metabolic signalling and insulin resistance. Nat Rev Endocrinol. 2014;10(12):723–36. Epub 20141007. doi: 10.1038/nrendo.2014.171. PubMed PMID: 25287287; PubMed Central PMCID: PMC4424797. 10.1038/nrendo.2014.171 PMC4424797 25287287

[30] Newgard CB, An J, Bain JR, Muehlbauer MJ, Stevens RD, Lien LF, et al. A branched-chain amino acid-related metabolic signature that differentiates obese and lean humans and contributes to insulin resistance. Cell Metab. 2009;9(4):311–26. 10.1016/j.cmet.2009.02.002. PMC3640280 19356713

[31] Pan Y, Xue F, Wang S, Zhong B, Zhan Y, Wang Q, et al. A real-world study of polyenyl phosphatidylcholine in the management of patients with metabolic dysfunction-associated fatty liver disease in China clinical practice. Front Med (Lausanne). 2025;12:1610083. 10.3389/fmed.2025.1610083. PMC12457410 41001365

[32] Metwally E, Burton L, Mirza I, Morsy MH, Scichilone G, Mostafa A, et al. Adiposome Lipidomic Shifts Following Bariatric Surgery in Association With Weight Loss and Cardiometabolic Benefit. J Clin Endocrinol Metab. 2026;111(4):964–79. 10.1210/clinem/dgaf586. PMC13017757 41137449

[33] Lee G, Park YS, Cho C, Lee H, Park J, Park DJ, et al. Short-term changes in the serum metabolome after laparoscopic sleeve gastrectomy and Roux-en-Y gastric bypass. Metabolomics. 2021;17(8):71. 10.1007/s11306-021-01826-y. 34355282

[34] Wahlström A, Aydin Ö, Olsson LM, Sjöland W, Henricsson M, Lundqvist A, et al. Alterations in bile acid kinetics after bariatric surgery in patients with obesity with or without type 2 diabetes. EBioMedicine. 2024;106:105265. 10.1016/j.ebiom.2024.105265. PMC11345581 39096744

[35] Tian J, Huang S, Sun S, Ding L, Zhang E, Huang W. Bile acid signaling and bariatric surgery. Liver Res. 2017;1(4):208–13. 10.1016/j.livres.2017.12.007. PMC6051716 30034914

[36] Zheng X, Chen T, Zhao A, Ning Z, Kuang J, Wang S, et al. Hyocholic acid species as novel biomarkers for metabolic disorders. Nat Commun. 2021;12(1):1487. 10.1038/s41467-021-21744-w. PMC7935989 33674561

[37] Narath SH, Mautner SI, Svehlikova E, Schultes B, Pieber TR, Sinner FM, et al. An untargeted metabolomics approach to characterize short-term and long-term metabolic changes after bariatric surgery. PLoS One. 2016;11(9):e0161425. 10.1371/journal.pone.0161425. PMC5008721 27584017

[38] Ionut V, Burch M, Youdim A, Bergman RN. Gastrointestinal hormones and bariatric surgery-induced weight loss. Obesity (Silver Spring). 2013;21(6):1093–103. 10.1002/oby.20364. PMC4423817 23512841

[39] Poitou C, Perret C, Mathieu F, Truong V, Blum Y, Durand H, et al. Bariatric Surgery Induces Disruption in Inflammatory Signaling Pathways Mediated by Immune Cells in Adipose Tissue: A RNA-Seq Study. PLoS ONE. 2015;10(5):e0125718. 10.1371/journal.pone.0125718. PMC4418598 25938420

[40] Hoggard N, Cruickshank M, Moar KM, Bashir S, Mayer CD. Using gene expression to predict differences in the secretome of human omental vs. subcutaneous adipose tissue. Obes (Silver Spring). 2012;20(6):1158–67. 10.1038/oby.2012.14. Epub 20120128. 22286531

[41] Kerr AG, Wang Z, Wang N, Kwok KHM, Jalkanen J, Ludzki A, et al. The long noncoding RNA ADIPINT regulates human adipocyte metabolism via pyruvate carboxylase. Nat Commun. 2022;13(1):2958. 10.1038/s41467-022-30620-0. PMC9135762 35618718

[42] Gonzalez-Plaza JJG-RC, Garcia-Serrano S, Rodriguez-Pacheco F, Garrido-Sanchez L, Santiago-Fernandez C, Garcia-Arnes J, Moreno-Ruiz FJ. Rodriguez-Cañete A, Garcia-Fuentes E. Transcriptional profiling of subcutaneous adipose tissue of morbidly obese subjects before and after 2 years of bariatric surgery. Gene Expression Omnibus (GEO); 2017.

[43] Petrus P, Mejhert N, Corrales P, Lecoutre S, Li Q, Maldonado E, et al. Transforming growth factor-β3 regulates adipocyte number in subcutaneous white adipose tissue. Cell Rep. 2018;25(3):551–60.e5. 10.1016/j.celrep.2018.09.069. 30332637

[44] Liu Y, Aron-Wisnewsky J, Marcelin G, Genser L, Le Naour G, Torcivia A, et al. Accumulation and Changes in Composition of Collagens in Subcutaneous Adipose Tissue After Bariatric Surgery. J Clin Endocrinol Metab. 2016;101(1):293–304. 10.1210/jc.2015-3348. 26583585

[45] Ortega FJ, Mercader JM, Moreno-Navarrete JM, Nonell L, Puigdecanet E, Rodriquez-Hermosa JI, et al. Surgery-Induced Weight Loss Is Associated With the Downregulation of Genes Targeted by MicroRNAs in Adipose Tissue. J Clin Endocrinol Metab. 2015;100(11):E1467–76. 10.1210/jc.2015-2357. 26252355

[46] Dantas WS, Roschel H, Murai IH, Gil S, Davuluri G, Axelrod CL, et al. Exercise-Induced Increases in Insulin Sensitivity After Bariatric Surgery Are Mediated By Muscle Extracellular Matrix Remodeling. Diabetes. 2020;69(8):1675–91. 10.2337/db19-1180. PMC7372074 32409493

[47] Kovac L, Gancheva S, Jähnert M, Sehgal R, Mastrototaro L, Schlensak M, et al. Different effects of bariatric surgery on epigenetic plasticity in skeletal muscle of individuals with and without type 2 diabetes. Diabetes Metab. 2024;50(5):101561. 10.1016/j.diabet.2024.101561. 38977261

[48] Kristensen MM, Jensen LJ, Hansen CN, Helge JW. F D. Weight loss following Roux-en-Y gastric bypass surgery leads to changes in the expression of several miRNAs and their predicted target genes in skeletal muscle. Gene Expression Omnibus (GEO). 2024.

[49] Park JJ, Berggren JR, Hulver MW, Houmard JA, Hoffman EP. GRB14, GPD1, and GDF8 as potential network collaborators in weight loss-induced improvements in insulin action in human skeletal muscle. Physiol Genomics. 2006;27(2):114–21. 10.1152/physiolgenomics.00045.2006. 16849634

[50] Häsler R. miRNA expression data from primary human tissue (gastric mucosa). Gene Expression Omnibus (GEO); 2016.

[51] Vomhof-DeKrey EE, Singhal S, Singhal SK, Stover AD, Rajpathy O, Preszler E et al. RNA Sequencing of Intestinal Enterocytes Pre- and Post-Roux-en-Y Gastric Bypass Reveals Alteration in Gene Expression Related to Enterocyte Differentiation, Restitution, and Obesity with Regulation by Schlafen 12. Cells. 2022;11(20). 10.3390/cells11203283 PMC9601224 36291149

[52] Ben-Zvi D, Meoli L, Abidi WM, Nestoridi E, Panciotti C, Castillo E, et al. Time-dependent molecular responses differ between gastric bypass and dieting but are conserved across species. Cell Metab. 2018;28(2):310–23.e6. 10.1016/j.cmet.2018.06.004. PMC6628900 30043755

[53] Pinhel MAS, Noronha NY, Nicoletti CF, de Oliveira BAP, Cortes-Oliveira C, Pinhanelli VC et al. Changes in Global Transcriptional Profiling of Women Following Obesity Surgery Bypass. Obes Surg. 2018;28(1):176 – 86. 10.1007/s11695-017-2828-x. 28735374

[54] Hulsmans M, Geeraert B, De Keyzer D, Mertens A, Lannoo M, Vanaudenaerde B, et al. Interleukin-1 receptor-associated kinase-3 is a key inhibitor of inflammation in obesity and metabolic syndrome. PLoS ONE. 2012;7(1):e30414. 10.1371/journal.pone.0030414. PMC3260289 22272346

[55] Berisha SZ, Serre D, Schauer P, Kashyap SR, Smith JD. Changes in whole blood gene expression in obese subjects with type 2 diabetes following bariatric surgery: a pilot study. PLoS ONE. 2011;6(3):e16729. 10.1371/journal.pone.0016729. PMC3053356 21423737

[56] Rashid M, Al Qarni A, Al Mahri S, Mohammad S, Khan A, Abdullah ML, et al. Transcriptome Changes and Metabolic Outcomes After Bariatric Surgery in Adults With Obesity and Type 2 Diabetes. J Endocr Soc. 2023;8(1):bvad159. 10.1210/jendso/bvad159. PMC10755185 38162016

[57] Kulkarni S. M R. Bariatric surgery mediated weight loss reduces breast cancer risk by reducing estrogen receptor alpha activity. Gene Expression Omnibus (GEO); 2022.

[58] van der Kolk BW, Heinonen S, White JW, Wagner A, Karppinen JE, Saari S, et al. Human skeletal muscle mitochondria responses to weight loss induced by bariatric surgery or lifestyle intervention. Acta Physiol (Oxf). 2026;242(2):e70150. 10.1111/apha.70150. PMC12783452 41507695

[59] Ahmed H, Fernandes MF, Abbas K, Synowsky SA, Shirran SL, Ajjan RA, et al. Quantitative proteomics identifies plasma protein alterations that associate with metabolic and thrombotic profile changes after bariatric surgery. Diabetes Obes Metab. 2025;27(5):2647–57. 10.1111/dom.16267. PMC11965011 40000393

[60] Wewer Albrechtsen NJ, Geyer PE, Doll S, Treit PV, Bojsen-Møller KN, Martinussen C, et al. Plasma Proteome Profiling Reveals Dynamics of Inflammatory and Lipid Homeostasis Markers after Roux-En-Y Gastric Bypass Surgery. Cell Syst. 2018;7(6):601–e123. 10.1016/j.cels.2018.10.012. 30528273

[61] Dadson P, Honka MJ, Suomi T, Haridas PAN, Rokka A, Palani S, et al. Proteomic profiling reveals alterations in metabolic and cellular pathways in severe obesity and following metabolic bariatric surgery. Am J Physiol Endocrinol Metab. 2025;328(3):E311–24. 10.1152/ajpendo.00220.2024. 39819027

[62] Lee H, Han J-A, Ahn H-S, Kwon SH, Kim K, Ryu S. Impact of diabetes on proteomic changes in circulating extracellular vesicles in individuals with obesity before and after bariatric surgery. Int J Obes. 2025;49(9):1874–81. 10.1038/s41366-025-01837-y. PMC12463671 40676151

[63] Pereira PR, Carrageta DF, Guerra-Carvalho B, Braga PC, Pereira J, Pereira SS, et al. Untargeted Urinary Proteomics Uncovers Nephroprotective and Systemic Adaptations after Obesity Surgery-Induced Weight Loss. J Proteome Res. 2026;25(1):119–30. 10.1021/acs.jproteome.5c00500. Epub 20251210. PMC12818830 41370325

[64] Gralka E, Luchinat C, Tenori L, Ernst B, Thurnheer M, Schultes B. Metabolomic fingerprint of severe obesity is dynamically affected by bariatric surgery in a procedure-dependent manner12. Am J Clin Nutr. 2015;102(6):1313–22. 10.3945/ajcn.115.110536. 26581381

[65] Han Y, Kim G, Ahn E, Jung S, Jung Y, Kim Y, et al. Integrated metagenomics and metabolomics analysis illustrates the systemic impact of the gut microbiota on host metabolism after bariatric surgery. Diabetes Obes Metab. 2022;24(7):1224–34. 10.1111/dom.14689. PMC9313881 35257467

